# Public institutions’ capacities regarding climate change adaptation and risk management support in agriculture: the case of Punjab Province, Pakistan

**DOI:** 10.1038/s41598-020-71011-z

**Published:** 2020-08-24

**Authors:** Nasir Abbas Khan, Qijie Gao, Muhammad Abid

**Affiliations:** 1 College of Humanities and Development Studies (COHD), China Agriculture University, Haidian District, No. 17 Qing Hua Dong Lu, Beijing, 100083 People’s Republic of China; 2 Centre for Climate Research and Development, COMSATS University Islamabad, Park Road, Tarlai Kalan, Islamabad, 45550 Pakistan

**Keywords:** Agroecology, Climate-change ecology, Grassland ecology, Climate sciences, Environmental social sciences, Natural hazards

## Abstract

Public institutions could play an important role in building agricultural resilience to climate change by providing information and technology support to farmers. This study takes the case of Pakistan to investigate the perspective and capacities of public institutions as well as to identify gaps in current institutional arrangements in dealing and managing climate change in the agriculture sector. For this purpose, 53 office bearers from thirteen public institutions in Punjab province of Pakistan were interviewed using a semi-structured questionnaire to collect data on climate knowledge, training, coordination, and resource availability. The study uses an index-based approach to calculate Institutional Capacity Indices (ICI) based on selected seven indicators. The results of the index analysis show that institutions have the least financial capacity, followed by lacking physical and human resources. Whereas results show high index value for perception and knowledge, indicating a good understanding of climate change at the institutional level. The overall ICI index value indicates a medium level capacity of institutions in dealing with climate change. Moreover, the study shows that gaps in management, non-availability of financial and physical resources, and lack of training are the key bottlenecks for limited adaptation support from public institutions. This study highlights the importance of reducing gaps so that institutions could play their role in building the resilience of the agriculture sector to climate change.

## Introduction

Agricultural systems in South Asia, including Pakistan, are highly sensitive and exposed to climate change and its impacts^1,2^. Particularly in Pakistan, where agriculture accounts for more than 40% of total labor forces and provides livelihood to almost two-thirds of the population, mainly resided in rural areas^3^. Over the past few decades, the country has become highly exposed to a range of climatic extremes, i.e., floods, droughts, heatwaves, climate-induced diseases, and pests; in result, agricultural production and livelihood of small farming communities have suffered a lot^4,5^. Further, low adaptive capacity and limited institutional access also make the agricultural systems more vulnerable to climatic risks^6^.

To avoid potential losses from climate change and related risks, climate change adaptation (CCA) and climate risk management (CRM) are considered as the important tools^7–9^. These farm-level strategies include changing crop variety and types, altering irrigation, conserving water, and diversifying crops^6,8,10^. Over the past decade, a substantial body of literature on climate change adaptation in Pakistan has now been available due to the increased focus of research on climate change and its interaction with agriculture sector^3,6,8^. One of the common findings in most of the studies is that agricultural adaptation is mainly private without a significant contribution from national or provincial governments or public institutions. Despite having climate change policy and action plans at federal and provincial level^11^, still, the current institutional frameworks are not aligned with support to approve such policies and plans^12^. Similarly, advisory and financial services provided by various public entities are not updated with the latest knowledge of climate change and hence often fail to support farmers’ adaptation needs^6,13^.

Public institutions can still play a significant role in shaping farmers’ adaptive capacity or resilience if equipped with updated knowledge and technologies^9,12^. For instance, updated and active institutions’ support (credit, advisory, and technical) may help farmers in acquiring updated information and cost-effective solutions to cope with climate-related uncertainties and reduce losses caused by climate change^9^. In contrast, the lack of institutional support could decrease farmers’ adaptive capacity and hence resilience to environmental hazards^12^. Globally, the rural governance system is dominated by formal institutions, comprised of public and private bodies having a top-down legislative hierarchy and systematic infrastructure to assist the farming communities^14,15^. Pakistan also has the well-established infrastructure of rural governance (mainly dominated by public institutions), which consists of the various departments established to deliver free-of-cost or low-cost services to farming communities^12^. These agricultural institutions are solely responsible for providing farmer support against every kind of emergency, including climatic hazards.

Considering the climate change vulnerability of Pakistan’s agriculture and significance of institutions’ support in CCA/CRM, it becomes a rationale to analyze the capacities of institutions working at the local level. Such assessment studies would prove great significance in both policy and practice aspects by identifying the existing gaps and potential solutions required for an extended CCA/CRM framework in agriculture^16,17^. In Pakistan, current research mainly focuses on farm level assessment of climate risks, impacts, and adaptation^3,5,6,8^. However, no part of any study (to our best knowledge) assessed institutional capacities regarding CCA/CRM in agriculture. Hence to fulfill this gap, the current research provides pioneering evidence from the leading agricultural province of Pakistan by evaluating institutions’ capacities regarding CCA/CRM in agriculture. The objectives of this study are (1) to assess the institution level understanding of climate change and its impact at the farm-level, (2) to analyze the agricultural institutions’ capacities regarding CCA/CRM, and (3) to identify the existing gaps and respective solutions in the current institutional arrangements.

### Theoretical background

The notion of institutional capacity, in conjunction with climate adaptation and risk management response, has gained significant prominence during recent years^16,17^. The literature on the institutional adaptive capacity of CCA/CRM ranges from flood disasters to water governance^10,18^ forest management to marine resources^15,19^. Climate change adaptive capacity, in general, refers to the resources or systems’ ability (physical or social) to respond to the existing or potential risks caused by environmental changes. Now the question emerges that what does adaptive capacity refer to when it relates to the institutions. There is no exact definition of institutional adaptive capacity in the literature. Gupta defines it as “the inherent characteristics of institutions that empower social actors to respond to short and long-term impacts either through planned measures or through allowing and encouraging creative responses from society both ex-ante and ex-post”^16^. Bierman defined institutions in climate governance context as the “formal and informal rules, rule-making systems, and actor networks at all levels of human society (from local to global) set up to steer societies towards preventing, mitigating, and adapting to global and local environmental change”^20^.

The concept of institutional adaptive capacity is mainly developed in the framework of climate change vulnerability and resilience, shaped by a system’s ability to modulate risk exposure and sensitivity^19^. Hence the adaptative capacity of a system is critical in determining its resilience; the more the adaptive capacity, the more the systems’ resilience^21^. More resilience indicates the more capacitated system with greater capability to respond to climate change risks. However, the theoretical perspectives define institutional adaptive capacity as “adaptive co-management,” “climate politics,” and “earth system governance”^16,19,20^. The current study follows the vulnerability and resilience framework and defines institutional capacity in terms of knowledge, financial, and technical resources, which enable effective response mechanisms dealing with CCA/CRM^19^.

## Research methodology

### Study area

This study was conducted in the Punjab province of Pakistan. Punjab was selected as the main study area because of its significant share in national agricultural production and subsequent vulnerability to climate change. Punjab contributes more than half of the country’s agricultural GDP by producing over 70% of the country’s total cereal yield^5^. The majority of the population still lives in rural areas and heavily relies on the agriculture sector for their subsistence^22^. In recent years, food security and livelihood of the rural population are under risk due to the negative impacts of extreme climatic events on crops in the province^1,3^. The frequency and intensity of extreme climatic events, i.e., floods, droughts, heatwaves, and wind storms, have been increased over the past decade^3,5^. During the last decade, Punjab has witnessed five major floods, which have caused fatalities and severe economic losses. The flood of 2010 alone severely affected the 11 districts of this province and destroyed two million hectares of unharvested crops, causing overall economic damage equivalent to USD 10 billion^4,23^. Drought and water shortages, on the other hand, significantly affected crop yields in the province^24^. Therefore, the province does need the provincial and federal government to act wisely to support the local adaptation of agricultural systems to climate change and related events.

### Sampling and data collection

A multi-stage sampling approach (MSS) was used to select the respondents from the study area. The rationale to employ such an approach was due to the varying hierarchical level of the institutions (from subdistrict to district, respectively, the second and third-level administrative units of local governance structure in Pakistan). The literature recommends the use of such an approach when the population is distributed at various levels. Then the sample is selected by choosing the respondent from each stage^25^. The key advantages of using MSS include flexibility in deciding the number of stages and method and the number of selecting sampling units from each stage, which make this approach more convenient in meeting survey reequipment^26^. However, time and cost are reported disadvantages of the MSS approach^25^. Hence following Abid^12^, we have selected the respondents involving four stages.

In the first stage, the Punjab province was selected as the main study area due to its significant agriculture sector and climate change vulnerability. In the second stage, 13 most relevant agricultural institutions were shortlisted in a consultative meeting with the director of agriculture (extension), considering their responsibilities and community support. The list of selected institutions with the description of provided services is given in Table 1. In the third stage, each of the chosen institutions was contacted for the allocation and availability of relevant key informant nominated by the director DoAE. In the fourth and final stage, a total of 53 officials were interviewed by face to face meetings. A primary questionnaire survey was used to obtain officials’ response, on the preselected indicators of institutions’ capacities (Table 2). The data collection was completed during the months of May–June 2019. After collecting data on selected dimensions, descriptive statistics and cross-tabulations were used to describe the data. Further, an index-based approach was used to present and compare institutions’ capacity across different level resources and types of institutions.

**Table 1:** Details of selected public sector agriculture institutions.

Institution name	Type of services	Operational level
1. Directorate of Agriculture (Extension) (DoAE)	Advisory services regarding farm operations	Sub-district
2. On-Farm Water Management (OFWM)	Watercourse improvement and subsidized farm implements including water-saving technologies	Sub-district
3. Farm Training and Adaptive Research (FTAR)	The experimental station, farmers’ training regarding technology adoption	District
4. Crop Reporting Services (CRS)	Crop yield, cultivated area, and diseases, insect, and pest attacks	District
5. Biological Control Laboratory (BCL)	Insect control, inspection, advisory, and training	District
6. Pest Warning nd Quality Control of Pesticides (PWQCP)	Advisory regarding Pest control, inspection, farmer training	District
7. Punjab Seed Corporation (PSC)	Seed sales, advisory services, seed quality testing	Sub-district
8. Soil and Water Testing Laboratories (SWTL)	Soil and water testing services, fertilizers advisories	District
9. Directorate General Agriculture (Field) (DoAF)	Land leveling, soil–water conservation, and drilling of Tube-wells)	District
10. Directorate of Agriculture (Economics nd Marketing) (DoAEM)	Agriculture marketing service, farmers training, and capacity building	Sub-district
11. Pakistan Agricultural Storage and Services Corporation (PASSC)	Public marketing of farm commodities	District
12. Punjab Irrigation Department (PID)	Irrigation services, canal management	Sub-district
13. Agricultural Development Bank (ZTBL)	Credit services to the farmers	Sub-district

**Table 2:** Descriptions of selected indicators and their literature sources.

Indicator	Sub-indicators	Description	Literature source
Perception and knowledge	Climate change (CC) perception	Changes in local climate (temperature, rainfall)	^15,16,28,45–47^
	Perceived impacts of CC	Impacts of CC (floods, droughts, biological hazards)	^15,16,28,45–47^
	Knowledge of CCA/CRM practices	Knowledge regarding climate-smart practices	^19,28,41,45–47^
	Beliefs regarding CCA/CRM	Acknowledgement of CCA/CRM practices to avoid farm-level impacts of CC	^10,18,19,28,38,41,45^
Training and expertise	Expertise regarding CCA/CRM	Practical skills regarding farm-level CCA/CRM	^7,16,18,19^
	Working experience	Previous working experience regarding CCA/CRM	^16,18,45,48^
	Professional training	Formal training course attended regarding CCA/CRM	^7,16,18,28,45^
	Trained staff	Percentage of trained staff in institution	^16,18,19,28^
Human resources	Staff availability	Staff available for general operations	^10,16,19^
	HR training needs	Whether staff needing training regarding CCA/CRM	^16,19,28,45^
	Staff Availability for CCA/CRM	Staff available for CCA/CRM related operations/emergencies	^16,19,29^
Plans and priorities	Institutional priority	CC is an important concern for the institutions	^15,17,29^
	Emergency planning	Emergency response planning for extreme events	^10,16,40^
	Past programs	Institutions carried out initiative/programs regarding CCA/CRM	^10,17,45^
	Current programs	Ongoing initiative/programs regarding CCA/CRM	^10,17,45^
	Future programs	Project in pipelines regarding CCA/CRM	^10,16,45,49^
Coordination and collaboration	Intra-institutional coordination	Coordination within the same institutions (research-field)	^10,16,17,19,28,45,47^
	Community coordination	Coordination/interaction with the farming communities	^10,15–18,29^
	Inter-institutional collaboration (Pub)	Collaboration with the other public sector’s institutions	^10,15,17,28,29,47,50^
	Inter-institutional collaboration (Pvt)	Collaboration with the private sector’s institutions	^15,17,18,28,29,46,50^
Financial resources	Funds availability	Any financial support being provided for CCA/CRM	^7,16,19,28,45–47^
	Funds sufficiency	Sufficiency of available finance in terms of meeting challenges	^7,16,19,28,45–47^
	Funds requirement	Current availability respect to actual requirement (percentage)	^7,16,19,28,45–47^
Physical resources	Machines/equipment availability (general)	Sufficiency of available machines/equipment for general operations	^15,19,47^
	Machinery/Equipment requirement	Extent of availability with respect to actual requirement (percentage)	^15,16,19^
	Machines/Equipment availability (CCA/CRM)	Sufficiency of available machines/equipment for CCA/CRM related operations and emergencies	^15,16,19^

### Institutional capacities assessment

Despite the critical significance of institution-led climate governance, literature shows little evidence regarding institutional capacities analysis. Therefore the methodologies regarding institutional capacity assessment remain at the evolving stage^14,19^. The most quoted approach in the literature is regarded as ACW (Adaptive Capacity Wheel), a framework to assess institutional adaptive capacities dealing with environmental challenges^16^. The ACW approach discusses six key dimensions to evaluate climate change governance (i.e., institutions’ learning capacity, human and financial resources, variety, leadership, and fair governance). Similarly, Institutional Analysis and Development (IAD) is another framework related to institutional capacities assessment, which mainly lies in the concept of action situation (level of interaction within institutions)^27^. However, these frameworks have certain practical limitations while choosing adaptive capacity determinants, in a specific context and study nature^19^.

In terms of empirical studies, a four indicator-based approach (collaboration, financial, technology, and information) was employed by Denny^28^, who assessed the institutional capacities of the Cambodian health and water sector regarding climate change. Bettini has also analyzed capacities of the Australian water governance by evaluating the stakeholders’ ability to learn, decide, and act^18^. Similarly, Social Network Analysis (SNA) is another widely used method to assess institutional capacities^15,29^, which, however, mainly focusses on the collaboration and coordination dimensions within an institutional hierarchy. Considering the literature gaps and existing empirical studies, we have found a seven indicator-based approach to assess the institutions’ capacities dealing with CCA/CRM in agriculture.

#### Index-based capacity assessment

In general, methods dealing with the adaptive capacity of the agricultural system vary depending on the intentions or goals, nature of assessment (local, regional or sectoral comparison), and its determinant factors^30^. In such studies, indicator-based assessment is considered as the most accepted method to precisely measure the agricultural adaptive capacity of climate change^21^. However, regarding institutional adaptive capacity, literature shows empirical studies based on simple qualitative, descriptive, and Likert scale assessment^15,28^, with rare evidence of indicator-based index approach^14^.

Studies advocate the use of indicator-based since it conceptualizes the theoretical concepts into a set of indicators or variables which serve as an operational representation of characteristics or qualities of a system^31,32^. Indicators-based index method generally requires a conceptual framework, study nature, goal, and context, which is followed by the selection of sub-indicators and variables under each component, data collection, and result aggregation^32–34^. The selected indicators are coded and combined into indices, which represent the multiple dimensions of adaptive capacity into a comparable range of values.

Despite broad recognition of the indicator-based indices across various disciplines, this approach has also received criticism due to certain limitations such as down and upscaling of the variables of various concepts and scales^31^; uncertainties in indicators selection^33,35^; data accuracy and accessibility^32^; robustness, and conceptual framing^30,32,35^. Notably, variable integration could emerge as a challenging task in this approach due to the specific functional relationship within the indicators and sub-indicators, varying with study context.

Nevertheless, despite various pitfalls, the indicator-based index approach is still considered as the most accurate method of adaptive capacity assessment even if the limitations, as mentioned above, are considered^21,30,31,33^. Hence finding this motivation, we have employed an index-based approach for institutional capacity assessment regarding CCA/CRM in agriculture.

#### Indicators selection and index calculation

Following an intensive literature review, seven key dimensions or indicators of institutional capacities were shortlisted in the current study. Each of the selected indicators was further divided into 3–5 sub-indicators concerning the main theme, comprising a total of 26 sub-indicators. The seven indicators included officials’ perception and knowledge of Climate change, and its impacts, training, and expertise, plans and priorities, coordination, human, financial, and physical resources of the institutions dealing with CCA/CRM. The detailed description of the selected indicators and sub-indicators with their reference source is given in Table 2.

To summarize the institutions’ capacities into selected seven indicators, the UNDP index aggregation method was used^36^. In this method, an index value was calculated, ranging between 0 and 1 (representing indicators strength from low to high). In the index calculation method, a normalization process is usually needed to make the variables’ value into a similar unit^31^. However, in the current study, all the values of sub-indicators were taken in percentages; hence no normalization process was required. Moreover, before index calculation, the sub-indicators, which had an adverse influence on respective indicators, were reversed^35^. Afterward, the indicators’ indices were calculated with the following method.

1 $$Index_{sd} = \frac{Sd - Smin}{{Smax - Smin}}$$

where *S*_*d*_ is the average of *ICI*-indicator, *S*_*min*_ is the minimum value of sub-indicators of the *ICI*-indicator, while *S*_*max*_ is the highest value of *ICI* sub-indicators. A total of seven indices were calculated, i.e., *PKI *(Perception and Knowledge Index), *TEI* (Training and Expertise Index), *HRI* (Human Resource Index), *PPI* (Plans and Priorities Index), *CCI *(Coordination and Collaboration Index), *FRI *(Financial Resources Index), and *PRI* (Physical Resources Index). After calculating the index for each indicator, a cumulative Institutional Capacity Index (*ICI*) was calculated as followed;

2 $$ICI = \frac{(PKI) + (TEI) + (HRI) + (PPI) + (CCI) + (FRI) + (PRI)}{7}$$

## Results and discussion

### Climate change and agriculture: an institutional perspective

In Pakistan, public institutions are considered among the key stakeholders in irrigated agriculture due to their importance in providing a range of services, i.e., surface irrigation, on-farm water management, pest and disease management, advisory, credit, and marketing services^12^. Hence it is pertinent to understand how these institutions perceive climate variability and its impacts in the study area.

Regarding observation on changes in climate, the majority of the office bearers reported substantial changes in temperature, rainfall, and cropping season expansion over the past 2 decades (Table 3). Notably, a significant increase in temperature and a decrease in rainfall is observed. Specifically, many respondents were of the view that summer seasons have become warmer. In contrast, monsoon rains, which account for two-thirds of the annual precipitation, has significantly decreased (shifting to late summer months). These observations are in line with the historical temperature and rainfall trends in the study area^1,3^. Further, respondents also indicated a variation in the duration of both *Rabi* (winter) and *Kharif *(summer) cropping seasons. An official from DoAE described that during the past few years, winter wheat cultivation is merged nearly a month to the summer season due to which the next crop faces delays in sowing and subsequent yield losses.

**Table 3:** Perceived climate changes and impacts at the farm level.

Climate change indicators	Impacts of climate change	
Rise in temperature	Heat intensity, over evaporation	Crop diseases, insect/pest attack, complex weeds
Declining rainfall	Reducing crop immunity	The resistance of insects and pests
Variation in cropping calendars	Drought, floods, hailing, wind storms	Shrinking ground and surface water resources
The shift in rainfall patterns	Irregularities in rivers and canals	Crop health, grain quality, yield losses
	Degradation in soil fertility and irrigation water quality	Reduced crop returns, decreasing farmland, migration, food insecurity

Source: (Institutional Survey, 2019).

In terms of climate-induced impact, the findings show that most of the effects reported are biophysical (droughts, floods, and water resources) and biological (insect, diseases, and weeds) in nature. Officials from PID and OFWM reported increasing water scarcity due to the reduced surface water flows and critical depletion of groundwater reserves that lead to the overall reduction in cultivated area under rice crop. Further, increased incidents of extreme temperature during early crop growth stages and intensive rainfall during harvesting seasons have severely affected rice yield. Heavy rain in late monsoon season leads to flooding in plain areas of Punjab and poses a severe threat to the sustainability of agriculture in the province.

Further, officials indicated that high temperatures and heatwaves have resulted in an increase in crop water requirements due to high evapotranspiration. Similarly, changing patterns of rainfall and extreme temperature events have increased the presence of fungal diseases, insect and weed attacks. Similar findings have been reported by a recent study showing a significant increase in the incidence and severity of climate-induced biological and biophysical risk in Pakistan^5^. Moreover, an official from DoAE reported a 100–150 kg/ha in general and 150–200 kg/ ha (in worst case scenario) reduction in wheat and rice yields due to increases in weed germination. Several respondents revealed that due to excessive use of insecticides and pesticides, aiming to control pests and diseases, the penetration of various harmful chemicals has alarmingly increased in both soil and water and resulted in degradation of water and soil quality.

In general, various respondents also highlighted the increase in unrest among farmers due to decreasing profit margins on account of the increasing cost of production and productivity decline due to climate change. Many farmers have been switched to non-farm businesses, and this lacking interest may further risk the national goal of sustainable food self-sufficiency and security.

### Institutional capacities regarding CCA/ CRM in agriculture

This study further analyzed the capabilities of agricultural institutions using seven indicators-based index approach. Results of the selected indicators are given in Table 4, which shows a medium level of preparedness and capacities of the selected institutions. Specifically, the results of each indicator are explained in the following.

**Table 4:** Institutional Capacities Index (*ICI*). Source: (Authors’ calculation based on institutional survey, 2019).

Indicator	Sub-indicators	Value	ICI Index
1. Perception and knowledge	Climate change (CC) perception	89.5	0.70
	Perceived impacts of CC	78.9	
	Knowledge of CCA/CRM practices	65.3	
	Beliefs regarding CCA/CRM	63.7	
2. Training and expertise	Expertise regarding CCA/CRM	39.7	0.55
	Working experience	31.1	
	Professional training	27.3	
	Trained staff	12.4	
3. Human resources	Staff availability (General)	31.1	0.44
	HR training needs	89.5	
	Staff availability (CCA/CRM)	26.1	
4. Plans and priorities	Institutional priority	36.8	0.66
	Emergency planning	35.5	
	Past programs	15.8	
	Current programs	42.1	
	Future programs	36.8	
5. Coordination and collaboration	Intra-institutional coordination	63.3	0.45
	Community coordination	32.7	
	Inter-institutional collaboration (Pub)	27.1	
	Inter-institutional collaboration (Pvt)	5.9	
6. Financial resources	Funds availability	15.8	0.36
	Funds sufficiency	11.8	
	Funds requirement	58.9	
7. Physical resources	Machines/equipment availability (general)	15.8	0.39
	Machinery/equipment requirement	48.9	
	Machines/equipment availability (CCA/CRM)	21.7	
Cumulative			0.51

#### Perception and knowledge

Literature shows that stakeholders’ perception and knowledge of climate change and its impact are among the key factors that define the level of intentions to make efforts regarding CCA/CRM^19^. These attributes allow an actor to formulate practices based on their knowledge and beliefs, which leads towards adequate risk management support^19,37^. Hence, officials’ perception and understanding of climate change impacts and risk management strategies were selected as the first indicator of institutional capacities assessment. Results (Table 4) show that overall, this indicator’s index maintained a good score, which is highest amongst all indicators. Specifically, most of the respondents had a significant perception of climate change and its induced impacts at the farm level. However, their knowledge and beliefs on adaptation strategies and their effectiveness are limited. Most of the respondents with negative beliefs about climate change adaptation were mainly from research and credit institutions. As reported by Farani^37^, a vigilant understanding of climate change is imperative to implement risk management mechanisms. Hence these findings imply to mainstream the climate change agenda across all agricultural institutions as they are part of the same institutional chain. This may lead to an equal understanding of climate-smart practices and hence improve institutions’ tendency to design and implement risk management mechanisms at the local level. A study reports similar findings on public health institutions, which also indicated the positive behavior of supervisors as an essential determinant of effective risk management services^38^.

#### Training and expertise

Institution’s technical resources, such as professional training and expertise, are also considered as crucial elements while dealing with climate hazards^19^. Such training helps office bearers to be well prepared and respond to catastrophes^39^. Current findings show that public institutions attained a medium level of training and expertise, as only 39% of the respondents possessed some knowledge regarding CCA/CRM. Similarly, two-third of the officials did not have any prior experience in climate risk management. Similarly, results show that only 12% of the officials received appropriate training related to CCA and CRM. However, one of the officials reported that since the last few years, some understanding of climate change had been developed at their department, and more officials are being invited for climate change-related training. Low training and expertise of agricultural office bearers in dealing with climatic risks may be translated into little support from public institutions to farming communities and hence may further increase the vulnerability of agriculture. Roosli^39^ was also of the view that skilled human resource is a pivotal attribute of institutions’ risk management capacity, as they have exceptional ability to provide technical aid to the disaster-prone communities by integrating and effectively using available resources. Fideldman^19^ has also raised the importance of staff’s skills in terms of integrating and implementing knowledge and mobilizing available resources against the environmental uncertainties. Further, professional knowledge and expertise not only improve the emergency response against climatic catastrophes but also improve the farmers’ and peers’ skills^39^.

#### Human resources

According to the Gupta’s Adaptive Capacity Wheel (ACW) framework, human resource has critical significance in determining the institutions’ abilities while dealing with climate risks^16^. Following ACW, human resources were also chosen as an indicator to assess institutional capacities. According to the findings, the *HR* index of the institutions reported a deficient value of 0.44. Sub-indicators further revealed that only 31% of institutions had sufficient human resources, and particularly only 26% of the institutions had adequate human resources to meet the operational requirement dealing with risk management emergencies. Officials from DoAE, OFWM, and PID indicated a severe shortage of skilled human resources to meet climate change challenges in the field operations. An official from PID described that, in case of any extreme climate event such as canal breakage, windstorm, or extreme hailing, sometimes quick response and technical support was not provided or possible due to limited skilled human resources.

These findings revealed that lack of human resources in public institutions might lead to limited risk management support and hence may further increase the vulnerability of farming communities to climate change. These results are supported by a study conducted in Congo, where forest institutions lacked in human resources in terms of climate change response^15^. Gupta was also of this view that institutions with adequate human resources have a greater ability to mobilize climate change adaption and risk management processes in agriculture. These findings conclude that sufficient human resources in public institutions are the prerequisite of active risk management support.

#### Plan and priorities

Institutions’ priorities, planning, and emergency response mechanism are widely reported as important factors in dealing with the environmental uncertainties^10,17,38^. According to our findings, public institutions attained a satisfactory score regarding this indicator (0.66). Specifically, one-third of the office barriers indicated climate change as an important agenda for their department. Similarly, in terms of programs and initiatives regarding climate change, 42% of the institutions reported that they are carrying related initiatives and programs. While one-third of the respondents were of the view that they are planning to add CCA/CRM in their priorities. Further, 35% of the institutions, mainly the field institutions such as PID, DoAE, DoAF, and CRS, indicated having an active emergency response mechanism dealing with climatic catastrophes.

Wenger^40^ reported that effective risk management response is closely associated with emergency planning within the institutions. Huq^10^, has also stressed the significance of defined objectives and plan among the key factors of successful implementation of adaptation and risk management response to flood disasters. Hence our study implies further strengthening the planning infrastructure by removing existing gaps, which will increase the institution’s abilities in dealing with environmental catastrophes.

#### Coordination and collaboration

A wide range of literature shows that coordination between different stakeholders is among the critical determinants of the institution’s adaptive and risk management capacities^15,16,28^ and often support collective action and decision making regarding climate change adaptation^15,41^. The *CCI *value of 0.45 showed that institutions had a minimal level of coordination with other stakeholders. For instance, in terms of community interaction, one-third of the institutions reported direct coordination with the farmers, indicating a reduced level of cooperation between the farmers and institutions. The officials who indicated coordination with farmers were mainly from the field institutions (PID, OFWM, DoAE, and SWTL). However, the research institutions had also acknowledged the significance of institution-community coordination. An official from a research institution (FTAR) stated that it is very pertinent for all institutions to have interactive communication with the farmers. However, most of the research institutions have a deficient level of community coordination, due to which most of the contingency plans and alerts (which usually go through the filed institutions) do not reach to the farmers timely. There is a need to develop such a communication system that could connect agricultural institutions and the farmers on a single communication platform.

In terms of inter-departmental collaboration, 27% of the respondents indicate that their respective institutions have a coordination mechanism with other public sector institutions. In comparison, merely 6% of them stated coordination with the private sector’s institutions. However, a decent level of coordination (63%) was indicated within the same institution. A minimal level of coordination, particularly between public and private institutions, is worrisome, as non-governmental bodies of Pakistan, which are already at the emerging stage, could face further marginalization^12^. Literature also advocates smooth coordination between the public and private organizations for effective adaptation and risk management support in agriculture^16^. Brown^15^ stated that a well-coordinated network between the actors of the same institution chain is critical for an active response to a challenge like climate change. Hence these findings conclude that a well-coordinated institutional setup may be more capable in coping with agricultural hazards.

#### Financial resources

Financial resources are also widely quoted among the significant determinants of institutional adaptive capacity^16,28^. Financial resources of the institution facilitate the actors’ preparedness and emergency response-ability towards natural disasters^42^. However, in the current study, the financial resources of the agricultural institutions were severely deficient (*FRI* 0.36). Findings revealed that only 15% of the institutions indicated funds availability for the CCA/CRM related operations. A significant majority of the officials (85%) reported the insufficiency of the financial resources available for climate change. Overall, a gap of nearly 40% was reported in terms of funds availability and requirement.

The respondents who indicated the availability of funds, particularly for CCA/CRM, were mainly from the research intuitions such as FTAR, SWTR, PWQP. Even though field institutions such as DoAE, DoAF, OFWM, PID have significant importance to carry community-level activities did not indicate enough financial support specified for CCA/CRM related operation. For instance, an official from DoAE reported a severe shortage of funds for launching emergency awareness campaigns and training seminars during the period of extreme weather events such as droughts, floods, heavy rains, and insect attacks. Due to financial constraints, such activities have been restricted to a few official visits or small gatherings in a few villages.

Apart from the field institutions, some credit providing institutions have also raised similar concerns. An official from ZTBL mentioned that in some situations when a cropping season faces unexpected yield losses due to rainfall or insect and disease attack. Farmers, particularly the smallholders, desperately need a loan to cultivate the next crop, and due to the unavailability of credit for such emergencies, the institution is unable to offer credit to these farmers.

Our findings are in line with the studies conducted in Cambodia^28^ and Cameron^15^, where institutions reported similar challenges while implementing climate response strategies. As argued by Gupta^16^, institutions’ financial resources are among the foremost determinants of effective adaptive and risk management in agriculture. These findings imply that the institutions, which are farmers’ first line of defense in an emergency, need to be strengthened in such a significant resource.

#### Physical resources

Access to adequate physical resources is considered as another critical component to define their role in supporting farmers to manage climate risks at the community level^15,43^. In terms of physical resources, availability of vehicles, machinery (harvesters, bulldozers, cranes), communication equipment, and hardware are considered for the capacity assessment of field and market institutions. In contrast, instruments, apparatuses, and laboratory equipment are considered for research institutions.

According to the results, the critical index value of the physical resources (0.39) indicates insufficient availability of infrastructure and physical resources in public institutions. Results of sub-indicators further revealed a vast gap (51%) between the availability and actual requirement of these resources. Only 21% of institutions indicated enough availability of machinery and hardware for extreme climatic conditions and emergencies. These figures are alarming as physical resources are pivotal elements while providing community support against catastrophes. Field intuitions, particularly the DoAE, DoAF, and PID, have indicated the critical shortage of these resources.

The officials from DoAE and PID have specifically indicated the lack of vehicles as the critical constraint limiting their efficiency while conducting the field operations. An official from DoAE revealed that most of the available vehicles are either very old or non-functional, which means filed staff has to wait hours and days to complete assigned field operations. Similar challenges were reported in terms of communication infrastructure as the officials from the DoAE highlighted a huge communication gap between farmers and their department due to the unavailability of contemporary communication tools. Previous studies^43^ have also reported similar findings of lacking logistic and communication resources and urged the provision of these resources for capacitated community support regarding natural disasters. In a nutshell, the physical resources of agricultural institutions are deficient in terms of meeting catastrophic challenges and seek serious consideration from concerned authorities.

### Institutional capacities across different types of institutions

**Figure 1:**
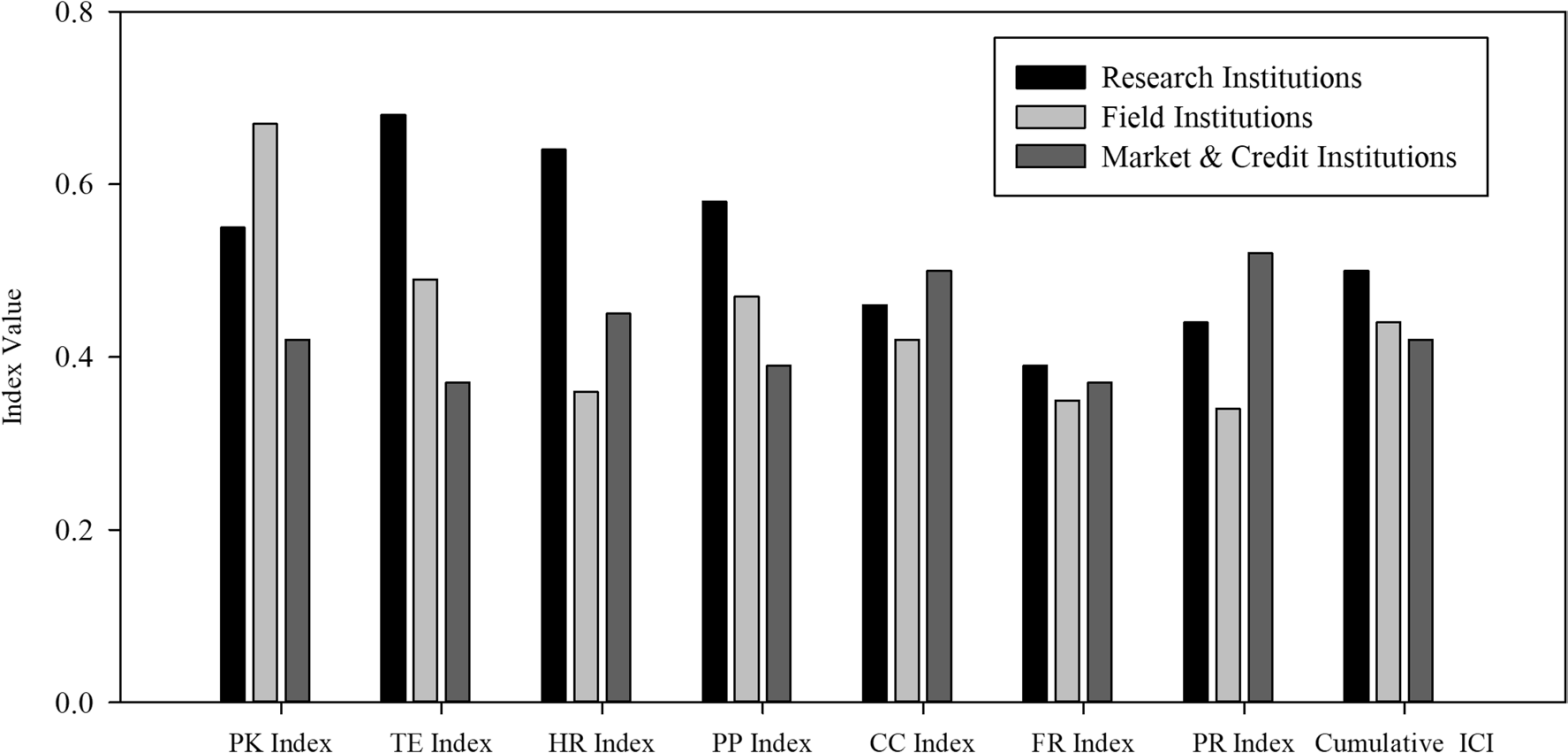
Institutional capacities index (*ICI*) across different categories of institutions.

To have a comprehensive understanding of institutional capacities across different types of Institutions,* ICI* was compared by categorizing the agricultural institutions into three categories, i.e., research, field, and market and credit institutions. Cumulative ICI values (Fig. 1) across these categories show that research institutions have attained higher index value, while credit and market, and field institutions are among the low capacitated institutions. The *ICI* values further show that perception and knowledge were high in case of field institutions, which could be due to their more field experience and interaction with farming communities. Such communication enables them to have a better understanding of climatic risks and farm level CCA/CRM practices. Moreover, financial resources showed the lowest value across all types of institutions. In terms of plans and priorities regarding CCA/CRM, research institutions maintained a higher index value.

In contrast, field, and credit and market institutions lacked in this indicator, highlighting the need for planning and prioritizing climate change agenda among these institutions. In terms of physical resources, which are regarded among the most critical resources, revealed alarming indications as both research and field institutions had a deficient amount of machinery and hardware resources. These findings imply that focus should be given to these institutions as they play a more crucial role (in terms of community support) when compared to credit and market institutions. Field institutions were also found lacking in terms of human resources, which could constraint the efficiency of these institutions in managing farm-level activities.

### Gaps and solutions

After exploring institutions’ capacities in the selected indicators, officials were asked to indicate existing gaps and related solutions, which are essential to increase the capacities in the context of climate governance and CCA/CRM in agriculture. The following gaps and solutions were identified and prioritized.

#### Need for an effective administrative mechanism

An effective administration and coordination mechanism has been listed as a top priority by most of the office-bearers to enhance the institutional capacity in managing climate risks. Officials also highlighted the importance of ensuring effective administrative mechanisms to implement and monitor the individual and collective performances in ongoing projects. That will improve the output of resources being invested at various levels. Fidelman and Madan^19^ have also indicated a sound administrative system among the critical components of the institution’s capacity dealing with CCA/CRM. Bettini raised the importance of constructing such a rule system that identifies accountability and defines boundaries and hierarchy in water management institutions^18^. Hence it is needed to develop or customize such institutional arrangements that are interactive, effectively administered, and target oriented.

#### Need for physical and financial resources

The second suggested measure is the provision of physical and financial resources required to support farm-level adaptation. Officials indicated that the current state of these resources is not enough to meet the institutional operational requirements to conduct CCA/CRM related operations. Brown has also identified similar gaps among the Congo’s forest institutions dealing with climate risk management^15^; however, Grecksch^14^ reported a higher level of physical and financial resources among the German institutions. Officials suggested that an appropriate amount of financial support should be specified for extreme climate events, along with emphasizing the need for communication and logistic resource. Literature also ranks these resources among the pertinent element of effective risk management^44^. The institutions equipped with such crucial resources would be more likely to overcome the climatic challenges. For instance, at the farm level, well-equipped institutions may have a better ability to reach farmers’ knowledge as well as technical requirements, to reduce the actual and potential losses. Similarly, the research institutions having contemporary technology apparatuses and instruments may create better innovation, i.e., climate-resilient farm inputs (seeds, water-efficient measures) that will ultimately reduce the farmers’ vulnerability of climate risks.

#### Need for professional training

Thirdly, a considerable portion of the respondent indicated the training need of staff regarding CCA and CRM. Institutions reported that human resources generally in the non-administrative and research positions, while particularly in field operations, are in much need of training. As indicated by Roosli that stakeholders may enhance the skilled humane resource by launching a series of training and disaster management programs that may lead to effective risk management response^39^. This study stresses that departmental training courses could be launched where indigenous and research knowledge could be integrated. Field staff should particularly be trained regarding emergency response in extreme climate events such as excessive rains, floods, wind storms. At the same time, the researcher’s skills should be enhanced in terms of the development of climate-smart practices and modeling farm-level risks and vulnerability.

#### Need for enhanced support

The last indicated challenge by the public institutions was the lack of support from the higher authorities. Institutions urged the need for a shared understanding and realization of agricultural vulnerability to climate change at both policy and higher administrative levels, which may put the energy into the local level. Similar capacity recommendations were identified by Brown^15^, where institutions reported a need for a common understanding between the stakeholders of forest communities for effective climate response.

## Conclusion and implications

Considering the vulnerability of the agriculture sector of Pakistan, the role of institutions support becomes imperative to effectively manage climate change risk and facilitate CCA/CRM at the farm level. This study assessed the capacities of the public institutions in Punjab province, providing support to the agriculture sector, using an index approach based on seven key indicators.

According to the institutional capacity index (ICI), institutional possessed a medium level of preparedness and capacities regarding CCA/CRM. However, a good understanding of climate change and its impacts on agricultural production had been observed. In line with the historical records, the majority of the respondents perceived an increase in average temperature, a decrease in rainfall, variation in cropping calendars, and increasing heatwaves in their respective areas. Similarly, the officials reported a growing negative impact of climate change on agricultural production due to climate-induced germination of complex weeds, frequent flooding, drought, incidents of diseases, and pest attacks. Such understanding is important in defining institutional strategy in dealing with climatic risks. However, study results show that institutional response to climatic threat is restricted due to lacking capacity of public institutions in terms of available financial, physical, human resource, and coordination.

Notably, the limited physical resources, i.e., communication tools and logistic facilities, often restrict field operations of the institutions working at farm-level. On the other hand, institutions involved in doing research and providing credit facilities also complained about having limited funds to manage climatic risks. Further, the majority of the officials did not receive any professional training on CCA or CRM and had limited capacity in supporting farmers to deal with climate change. These findings urge the need for staff training as technical resources are critical in enhancing farm-level CCA/CRM support.

Similarly, concerning financial resources, many of the institutions did not indicate financial support specified for CCA/CRM and reported a considerable gap in funds availability and requirement. These results imply that in the case of a vulnerable agriculture sector, lacking financial resources may emerge as a potential challenge to public institutions to provide risk management support. Similar gaps were identified in physical resources, as nearly half of institutions indicated the insufficient availability of hardware and machinery needed to meet their operational requirement. These results impulse the immediate need for the provision of the physical infrastructure to meet institutions’ requirements in terms of these crucial resources.

This study further identified and prioritized the existing gaps in the current institutional arrangement and suggested related measures to enhance institutions’ capacities by addressing these gaps. According to the findings, lack of effective administrative mechanisms, inadequate financial and physical resources, and lack of professional training were identified as significant gaps, which can be addressed by capacitating institutions in the mentioned areas. Notably, there is a need to develop an effective administrative system where the practical implementation of the programs is ensured. Moreover, to effectively address the climate-induced challenges in agriculture, a significant increase in financial and physical resources is required. Hence this study, based on these findings, seeks consideration from the policymakers, higher authorities to critically consider these gaps and solutions to ensure a well capacitated institutional support to the farming communities in Pakistan.

## References

[1] Ahmad A (2015). Handbook of Climate Change and Agroecosystems: The Agricultural Model Intercomparison and Improvement Project Integrated Crop and Economic Assessments, Part 2.

[2] Aggarwal P, Sivakumar MV (2010). Climate Change and Food Security in South Asia.

[3] Abid M, Scheffran J, Schneider UA, Ashfaq M (2015). Farmers’ perceptions of and adaptation strategies to climate change and their determinants: The case of Punjab province, Pakistan. Earth Syst. Dyn..

[4] Shah AA (2018). Flooding in Khyber Pakhtunkhwa: Gathering lessons learned and perceptions at the community level of the NGOs extended shelter intervention program. Int. NGO J..

[5] Khan NA, Gao Q, Iqbal MA, Abid M (2020). Modeling food growers’ perceptions and behavior towards environmental changes and its induced risks: Evidence from Pakistan. Environ. Sci. Pollut. Res. Int..

[6] Fahad S, Wang J (2018). Farmers’ risk perception, vulnerability, and adaptation to climate change in rural Pakistan. Land Use Policy.

[7] Nelson R (2010). The vulnerability of Australian rural communities to climate variability and change: Part II-Integrating impacts with adaptive capacity. Environ. Sci. Policy.

[8] Ashraf M, Routray JK, Saeed M (2014). Determinants of farmers’ choice of coping and adaptation measures to the drought hazard in northwest Balochistan, Pakistan. Nat. Hazards.

[9] Glaas E, Jonsson A, Hjerpe M, Andersson-Sköld Y (2010). Managing climate change vulnerabilities: Formal institutions and knowledge use as determinants of adaptive capacity at the local level in Sweden. Local Environ..

[10] Huq N (2016). Institutional adaptive capacities to promote ecosystem-based adaptation (EbA) to flooding in England. Int. J. Clim. Change Strat. Manag..

[11] Mumtaz M (2018). The national climate change policy of Pakistan: An evaluation of its impact on institutional change. Earth Syst. Environ..

[12] Abid M, Ngaruiya G, Scheffran J, Zulfiqar F (2017). The role of social networks in agricultural adaptation to climate change: Implications for sustainable agriculture in Pakistan. Climate.

[13] Khan NA, Qijie G, Ali S, Shahbaz B, Shah AA (2019). Farmers’ use of mobile phone for accessing agricultural information in Pakistan: A case of Punjab province. Cie. Rural..

[14] Grecksch K (2013). Adaptive capacity and regional water governance in north-western Germany. Water Policy.

[15] Brown HCP, Nkem JN, Sonwa DJ, Bele Y (2010). Institutional adaptive capacity and climate change response in the Congo Basin forests of Cameroon. Mitig. Adapt. Strat. Glob. Change.

[16] Gupta J (2010). The Adaptive Capacity Wheel: A method to assess the inherent characteristics of institutions to enable the adaptive capacity of society. Environ. Sci. Policy.

[17] Brown HCP, Smit B, Somorin OA, Sonwa DJ, Ngana F (2013). Institutional perceptions, adaptive capacity and climate change response in a post-conflict country: A case study from Central African Republic. Clim. Dev..

[18] Bettini Y, Brown RR, de Haan FJ (2015). Exploring institutional adaptive capacity in practice: Examining water governance adaptation in Australia. Ecol. Soc..

[19] Fidelman P, Tuyen TV, Nong K, Nursey-Bray M (2017). The institutions-adaptive capacity nexus: Insights from coastal resources co-management in Cambodia and Vietnam. Environ. Sci. Policy.

[20] Biermann, F., Michele M. Betsill, Joyeeta Gupta, Norichika Kanie, Louis & Lebel, D. L., *Heike Schroeder, and Bernd Siebenhüner. Earth System Governance: People, Places and the Planet. Science and Implementation Plan of the Earth System Governance Project. Earth System Governance Report 1, IHDP Report 20*. Bonn, IHDP. (2009).

[21] Eakin H, Luers AL (2006). Assessing the vulnerability of social-environmental systems. Annu. Rev. Environ. Resour..

[22] Khan NA, Qijie G, Sertse SF, Nabi MN, Khan P (2019). Farmers’ use of mobile phone-based farm advisory services in Punjab, Pakistan. Inf. Dev..

[23] Atif I, Mahboob MA, Waheed A (2015). Spatio-temporal mapping and multi-sector damage assessment of 2014 flood in Pakistan using remote sensing and GIS. Indian J. Sci. Technol..

[24] Xie H, Ringler C, Zhu T, Waqas A (2013). Droughts in Pakistan: A spatiotemporal variability analysis using the Standardized Precipitation Index. Water Int..

[25] Allen M (2017). Sampling Multisatege. The SAGE Encyclopedia of Communication Research Methods.

[26] Steel D (2011). International Encyclopedia of Statistical Science.

[27] Blomquist W, deLeon P (2011). The design and promise of the institutional analysis and development framework. Policy Stud. J..

[28] Dany V, Bowen KJ, Miller F (2015). Assessing the institutional capacity to adapt to climate change: A case study in the Cambodian health and water sectors. Clim. Policy.

[29] Romero-Lankao P, Hughes S, Rosas-Huerta A, Borquez R, Gnatz DM (2013). Institutional capacity for climate change responses: An examination of construction and pathways in Mexico City and Santiago. Environ. Plan. C Govern. Policy.

[30] Eriksen SH, Kelly PM (2007). Developing credible vulnerability indicators for climate adaptation policy assessment. Mitig. Adapt. Strat. Glob. Change.

[31] Birkmann J (2006). Indicators and criteria for measuring vulnerability: Theoretical bases and requirements. Meas. Vulner. Nat. Hazards Disast. Resil. Soc..

[32] Becker, D., Schneiderbauer, S., Forrester, J. M. & Pedoth, L. (CRED, Louvain, 2015).

[33] Binder CR, Feola G, Steinberger JK (2010). Considering the normative, systemic and procedural dimensions in indicator-based sustainability assessments in agriculture. Environ. Impact Assess. Rev..

[34] Asare-Kyei DK, Kloos J, Renaud FG (2015). Multi-scale participatory indicator development approaches for climate change risk assessment in West Africa. Int. J. Disast. Risk Reduct..

[35] Vincent K (2007). Uncertainty in adaptive capacity and the importance of scale. Glob. Environ. Change.

[36] UNDP. Human Development Reports. https://hdr.undp.org/en/. Accessed 28 Dec 2019 (2007).

[37] Farani AY, Mohammadi Y, Ghahremani F (2019). Modeling farmers’ responsible environmental attitude and behaviour: A case from Iran. Environ. Sci. Pollut. Res..

[38] Hilton T (2015). Perceived attitudes and staff roles of disaster management at CBOCs. Feder. Pract. Health Care Prof. VA DoD PHS.

[39] Roosli R, O’Brien G (2011). Social learning in managing disasters in Malaysia. Disast. Prev. Manag. Int. J..

[40] Wenger D, James T, Faupel C (1980). Disaster planning: An examination of disaster plans and public expectations. Disast. Beliefs Emerg. Plan..

[41] Grothmann T, Grecksch K, Winges M, Siebenhuner B (2013). Assessing institutional capacities to adapt to climate change: Integrating psychological dimensions in the Adaptive Capacity Wheel. Nat. Hazards Earth Syst. Sci..

[42] Jackson D (2011). Effective financial mechanisms at the national and local level for disaster risk reduction. United Nations Capital Dev. Fund.

[43] Madan A, Routray JK (2015). Institutional framework for preparedness and response of disaster management institutions from national to local level in India with focus on Delhi. Int. J. Disast. Risk Reduct..

[44] Madan S, Burman RR, Sharma JP, Sangeetha V, Iquebal MA (2015). Constraints faced in mobile based agro-advisory services and strategy for enhancing the effectiveness of mKRISHI. Indian Res. J. Extension Educ..

[45] FAO. Food and Agriculture Organization. Institutional capacity assessment approach for national adaptation planning in the agriculture sectors. https://www.fao.org/3/I8900EN/i8900en.pdf. (2018).

[46] Shakya, K. K. C., Gupta, N., Bull, Z, & Greene, S. Building institutional capacity for enhancing resilience to climate change: An operational framework and insights from practice. Oxford Policy Management. https://pubs.iied.org/X00194/. (2018).

[47] Kuhlicke C (2011). Perspectives on social capacity building for natural hazards: Outlining an emerging field of research and practice in Europe. Environ. Sci. Policy.

[48] Olesen JE (2007). Uncertainties in projected impacts of climate change on European agriculture and terrestrial ecosystems based on scenarios from regional climate models. Clim. Change.

[49] Pielke RA (1998). Rethinking the role of adaptation in climate policy. Glob. Environ. Change Human Policy Dimens..

[50] Olsson P, Folke C, Berkes F (2004). Adaptive co-management for building resilience in social–ecological systems. Environ. Manag..

